# Supplementary material for: Pol II degradation activates cell death independently from the loss of transcription
Source: bioRxiv. 2024 Dec 10:2024.12.09.627542. Preprint. [Version 1] doi: 10.1101/2024.12.09.627542 (PMC11661175; doi:10.1101/2024.12.09.627542)
Supplement: Supplementary file 1 [file media-1.pdf]

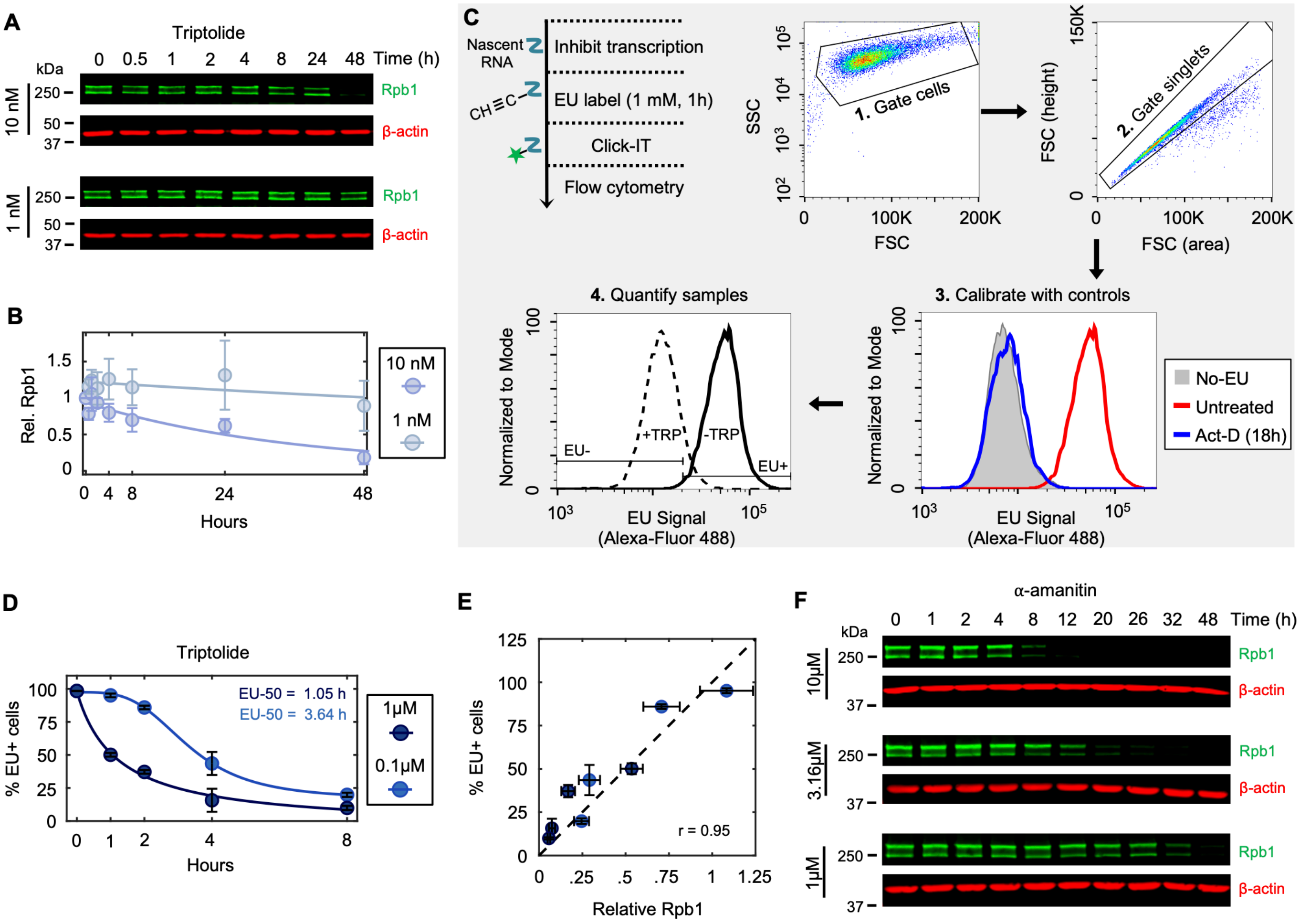

Fig. S1: Establishing kinetics of complete transcriptional inhibition. (**A**) Immunoblots of total Rpb1 in U2OS cells following exposure to low doses of triptolide (10 nM or 1 nM). Blots are representative of three independent biological replicates. (**B**) Quantification of Rpb1 levels shown in panel (A). Data are normalized relative to untreated levels. (**C**) Approach for measuring incorporation of 5-ethynyl uridine (EU) into newly synthesized RNAs using flow cytometry. Following labeling of nascent RNA via incubation with EU, cells are fixed, and labeled RNA is ligated to a fluorophore using click chemistry. A high dose (1 μM) and long incubation with the pan-RNA polymerase inhibitor actinomycin-D (Act-D) is used as a control to define the signal associated with no active transcription (EU-). (**D**) EU incorporation in U2OS cells before and after triptolide. Data are mean ± SD, n = 3. (**E**) Correlation between Rpb1 protein loss (quantified by immunoblot) and nascent RNA loss (quantified by EU incorporation) following exposure to 1 μM and 0.1 μM triptolide (at 1, 2, 4 and 8 hours post drug addition). Dashed line, x = y. Pearson correlation coefficient is shown. Data are mean ± SD, n = 3. (**F**) Immunoblots of total Rpb1 protein in U2OS cells following exposure to a dose range of α-amanitin. Blots are representative of three independent biological replicates.

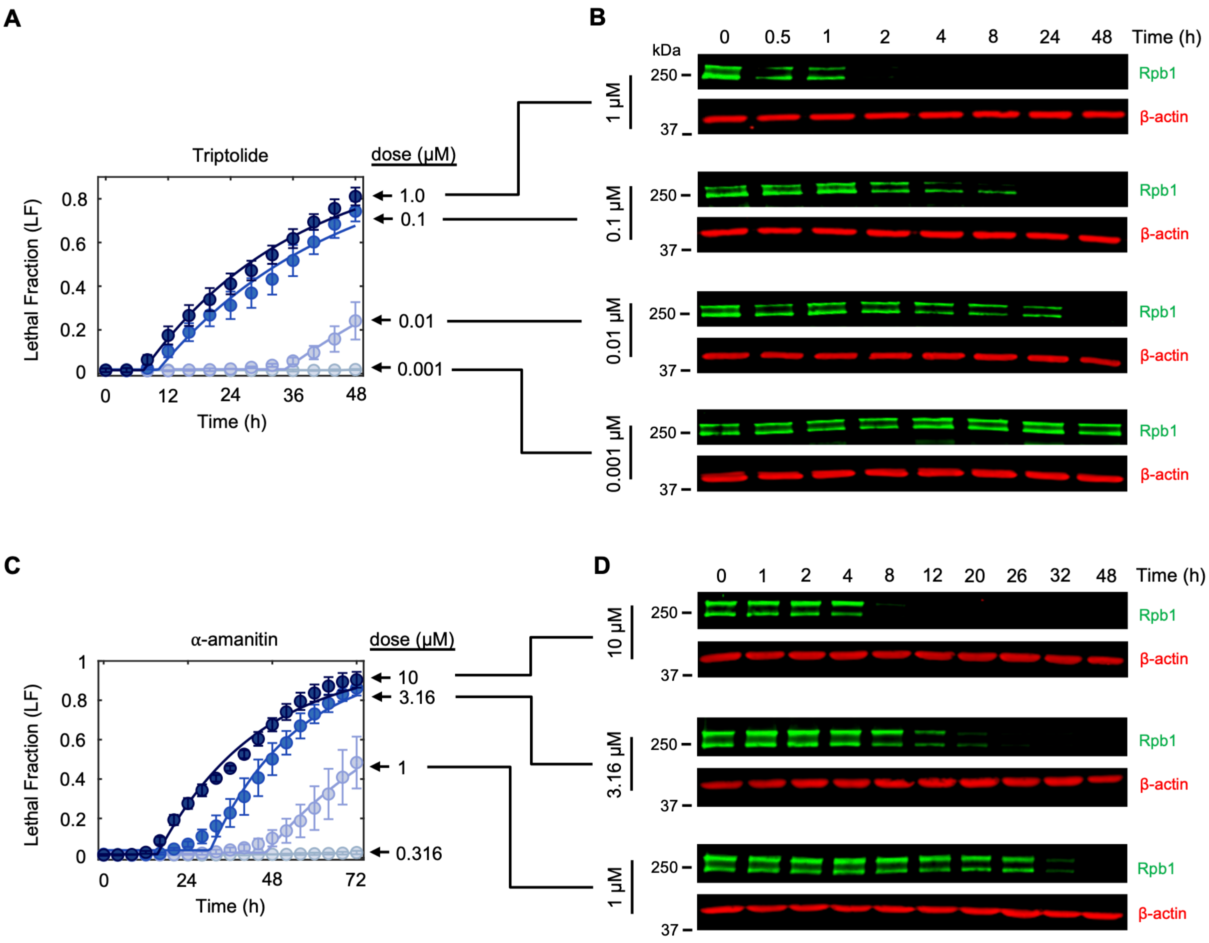

Fig. S2: Cell death kinetics for triptolide and α-amanitin across dose. (**A**) Lethal fraction kinetics in U2OS cells for triptolide across dose, measured in STACK. Data are mean ± SD, n = 3 independent biological replicates. (**B**) Immunoblots of Rpb1 protein levels in U2OS cells following exposure to triptolide across dose. Dose associations with (A) are shown. Blots are representative of three independent biological replicates. (**C-D**) As in (A-B), but for α-amanitin.

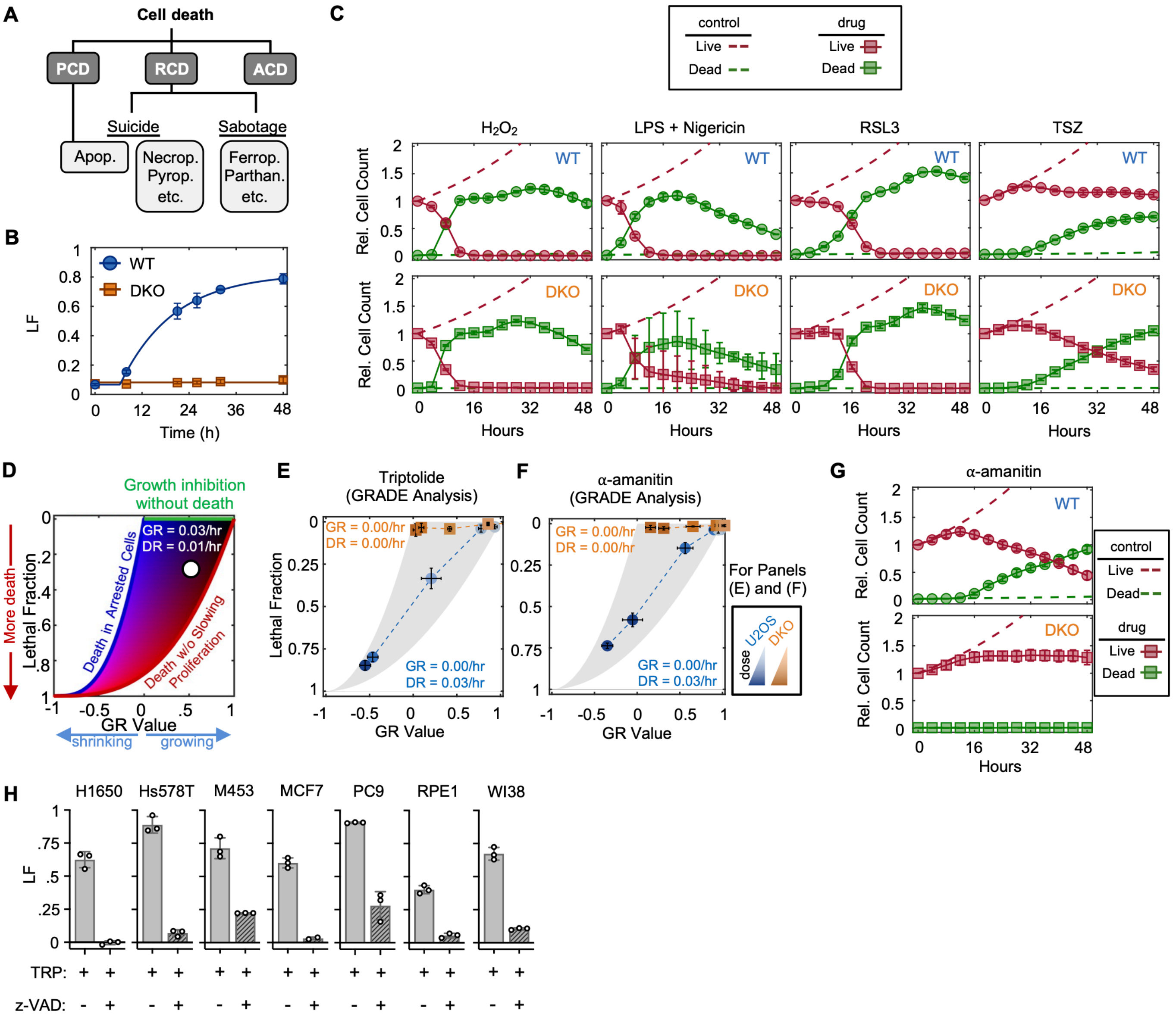

Fig. S3: Evaluation of cell death following Pol II degradation. (**A**) Cell death can be broadly classified into three groups: "Accidental cell death" (ACD) results from a stress that overwhelms cellular control mechanisms; "Programmed cell death" (PCD) occurs in normal development and tissue homeostasis; "Regulated cell death" (RCD) is executed by defined signaling events and effector molecules in response to exogenous stresses. RCD is further sub-divided into cell death pathways activated via signaling (cell "suicide") or those that take advantage of cellular activities but are not necessarily due to signaling (cell "sabotage"). (**B**) LF kinetics in U2OS (WT) and U2OS$^{BAX-/-BAK1-/-}$ (DKO) cells following exposure to the apoptotic agent ABT-737 (31.6 µM), measured in FLICK. (**C**) Live and dead cell kinetics following exposure to various non-apoptotic stimuli (Oxeiptosis, 316 µM H_2O_2; Pyroptosis, 0.2 µg/mL LPS + 31.6 µM Nigericin; Ferroptosis, 31.6 µM RSL3; Necroptosis, 10 ng/mL TNFα + 10 µM SM-164 + 50 µM z-VAD). Data collected using the STACK assay. (**D**) Schematic of the GRADE method. Drug-induced growth and death rates can be inferred from GR/LF plots. (**E**) GRADE-based analysis of triptolide at 48 hours. Inference of growth (GR) and death (DR) rates for the highest dose (1 µM) are shown for both genotypes. (**F**) As in (E) but for α-amanitin. **(G)** Same as (C), for 10 µM α-amanitin. (**H**) Lethal fraction kinetics for a panel of cell lines following 72-hour treatment with 1 µM triptolide, with- or without co-treatment with 50 µM z-VAD, measured using FLICK. (B, C, E, F, G, H) Data are mean ± SD, n = 3.

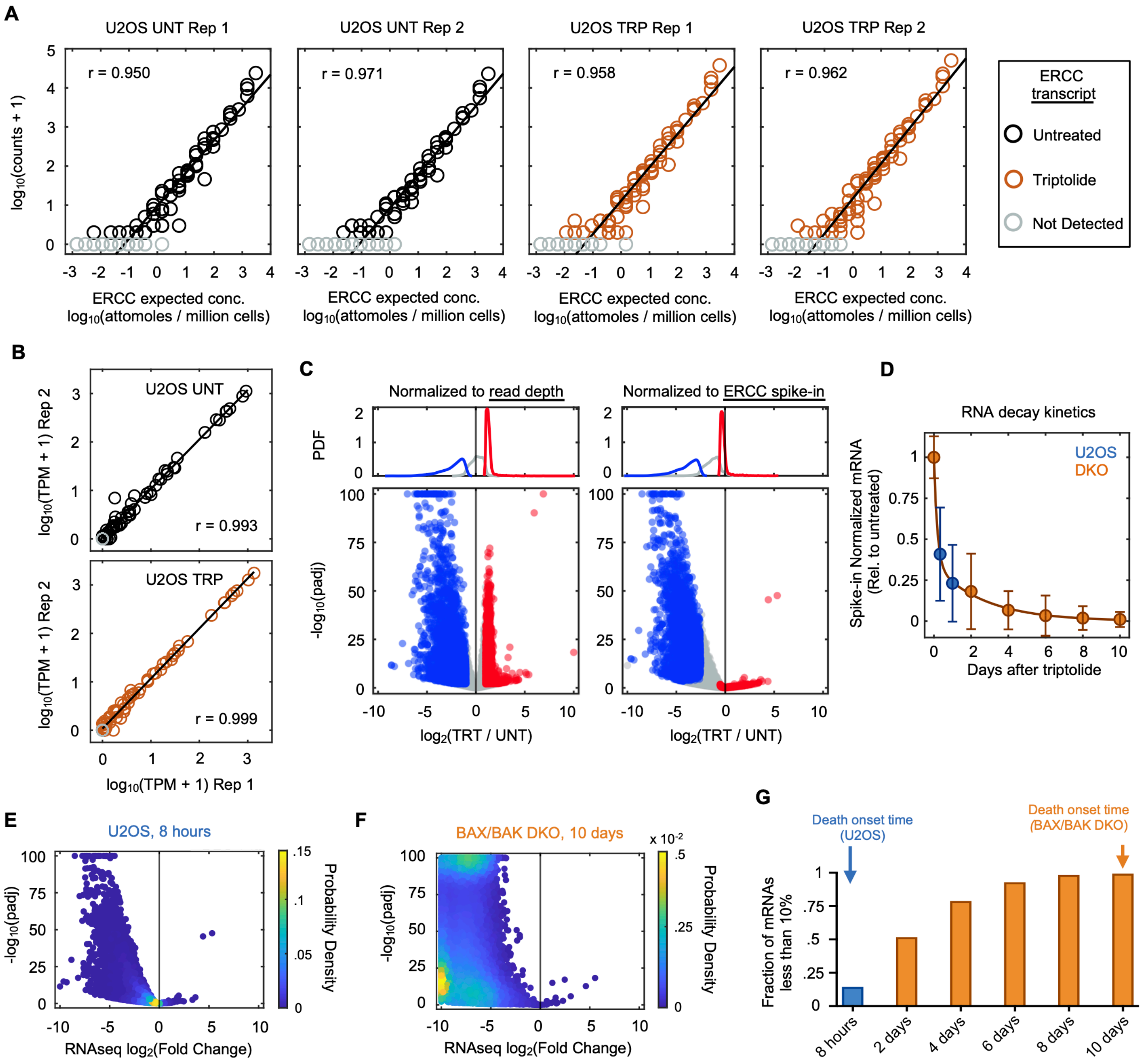

Fig. S4: Normalization of RNAseq data to polyadenylated ERCC spike-ins. (**A**) The expected molar amount of RNA for each of the 92 ERCC spike-in transcript standards compared to the empirically observed read counts, for replicates of U2OS RNAseq samples untreated (UNT) or treated with 1 µM triptolide (TRP) for 8 hours. Grey circles denote ERCC transcripts with zero counts. (**B**) Correlation between replicates for spike-in normalized transcripts per million (TPM). (**C**) Spike-in normalization is required to measure mRNA on an absolute scale. RNA-seq profiling of mRNA expression changes following exposure to 1 µM triptolide for 8 hours. (Left) mRNA expression normalized to read depth. (Right) mRNA expression normalized to ERCC spike-ins. Genes are colored based on read depth normalization: grey: no change; blue: down; red: up. (**D**) Decay kinetics for mRNAs in U2OS and BAX/BAK DKO (DKO) after 1 µM triptolide exposure for the indicated times. (**E**) Spike-in normalized fold changes in U2OS cells following 8 hours of 1 µM triptolide. (**F**) Spike-in normalized fold changes in *BAX/BAK* DKO cells following 10 days of 1 µM triptolide. (**G**) Fraction of mRNAs that have decayed beyond 90% of untreated steady-state abundance. U2OS (blue) and BAX/BAK DKO cells (orange) are shown, and their respective death onset times are noted.

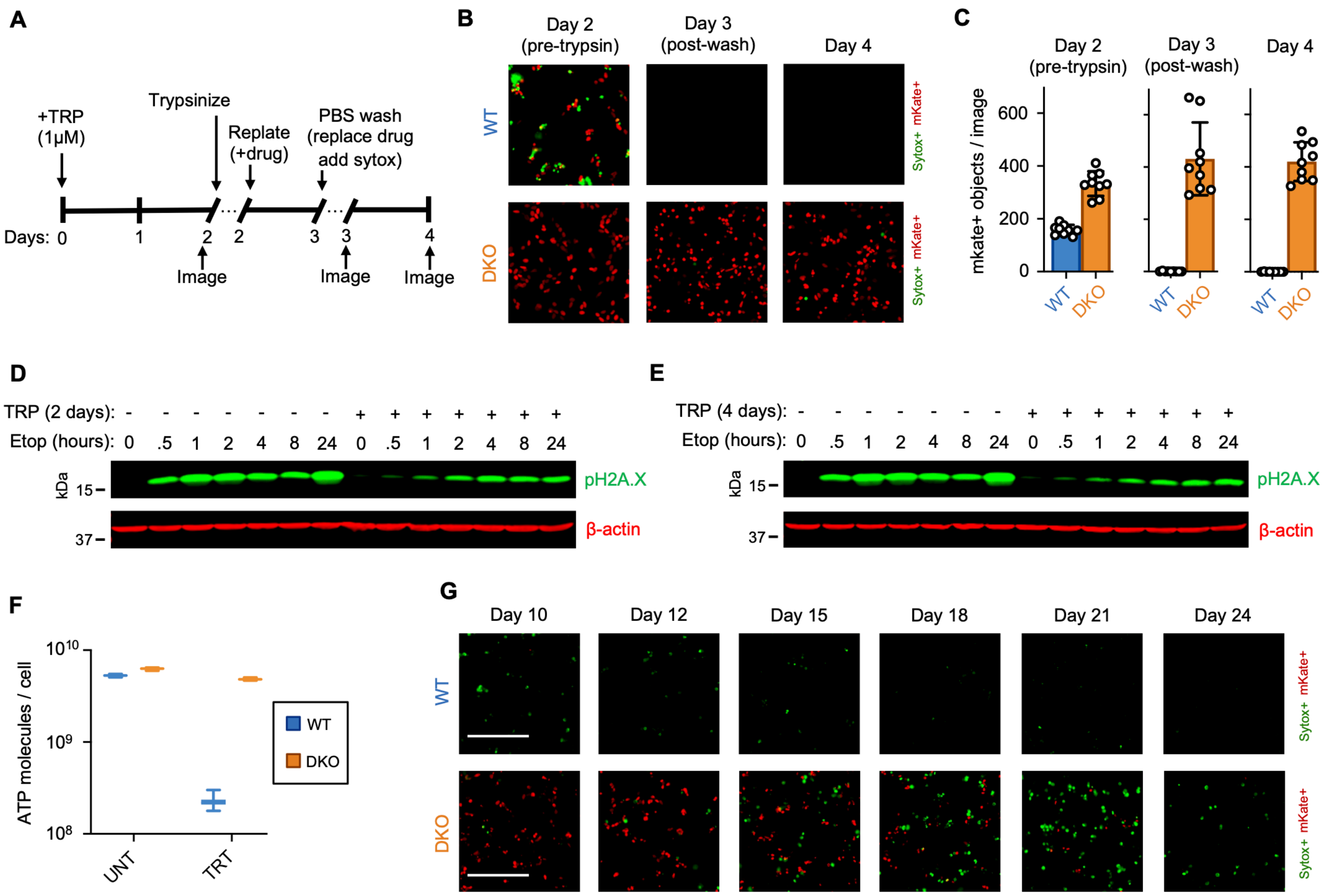

Fig. S5: Viability of *BAX/BAK* DKO cells during long-term exposure to triptolide. (**A**) Experimental overview for testing the ability of apoptotic deficient cells to reattach to plastic following trypsinization. (**B**) Representative images of triptolide treated cells before and after trypsinization and subsequent replating. (**C**) Quantification of mKate+ nuclei per image before and after trypsinization and subsequent replating, associated with (B). (**D**) Immunoblot depicting intact DNA damage response signaling in both untreated and triptolide-treated BAX/BAK DKO cells. Cells were first treated with or without 1 μM triptolide for 2 days to induce Pol II degradation, following by addition of 31.6 μM Etoposide to induce DNA damage. pH2A.X (S139) levels were monitored over time following Etoposide. (**E**) As in (D), except BAX/BAK DKO cells were exposed to triptolide for 4 days. (**F**) ATP molecules per cell following a 4-day treatment with 1 μM triptolide in U2OS or BAX/BAK DKO cells. ATP was measured using Cell Titer Glo, and cell number was normalized using FLICK. (**G**) Long-term survival of BAX/BAK DKO cells following 1 μM triptolide (associated with Fig. 1F). Representative images for three independent biological replicates is shown.

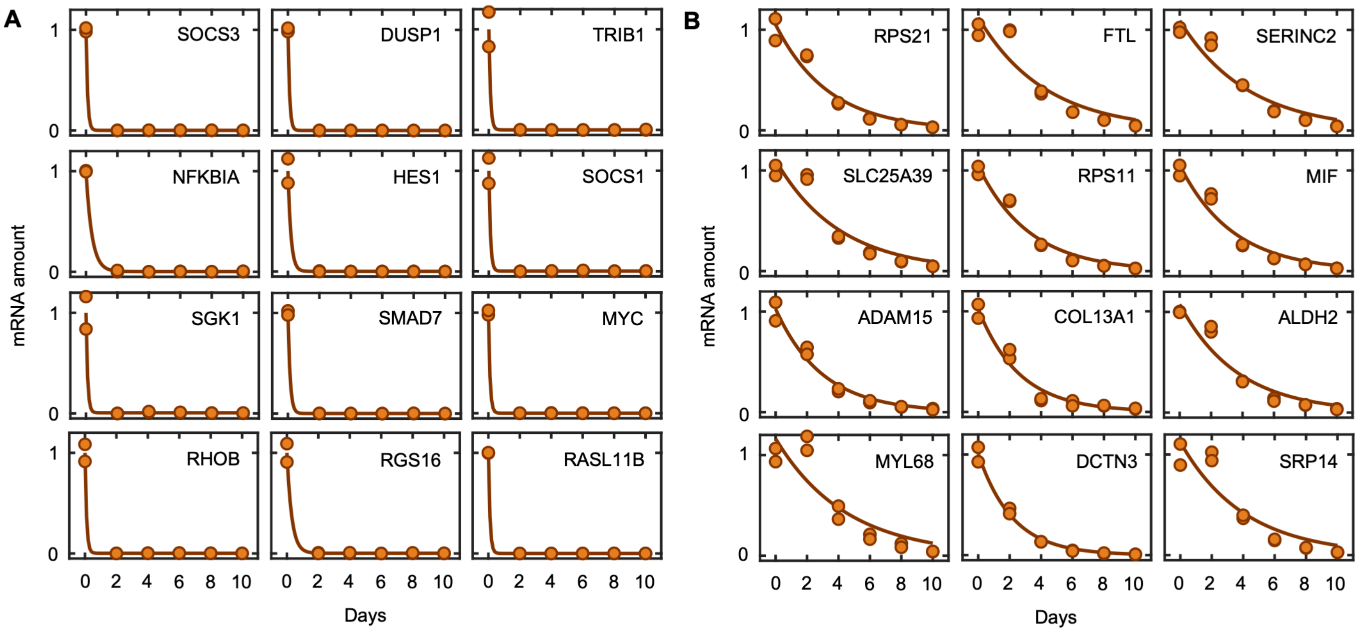

Fig. S6: Decay kinetics of short and long-lived transcripts. (**A**) mRNA decay kinetics in DKO cells following long-term exposure to 1 μM triptolide, measured using spike-in normalized RNAseq. 12 shortest half-life transcripts, defined from a meta-analysis of half-life measurements Agarwal and Kelley (2022). (**B**) As in (A), for the 12 longest half-life transcripts.

A

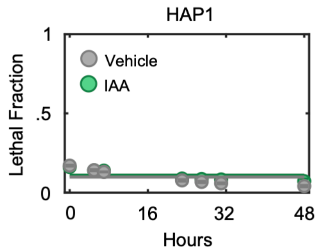

Fig. S7: Cell death in HAP1-RPB1-AID cells is not caused by auxin itself. (**A**) Lethal fraction kinetics in parental HAP1 cells following exposure to 500 μg/mL auxin (IAA, 3-indoleacetic acid) or vehicle, measured in FLICK. Data are mean ± SD, n = 3 independent biological replicates.

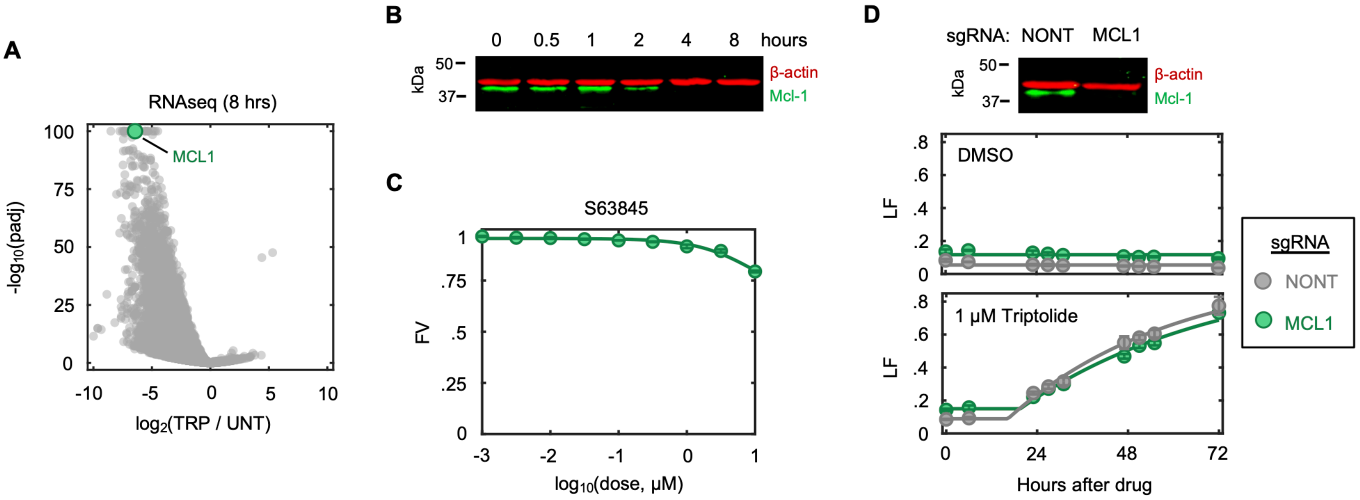

Fig. S8: Loss of Mcl-1 protein following transcriptional inhibition is not causal for apoptosis. (**A**) mRNA expression following 8-hour exposure to 1 μM triptolide in U2OS cells, highlighting the rapid loss of MCL1. (**B**) Immunoblot of Mcl-1 protein levels in U2OS cells following exposure to 1 μM triptolide. (**C**) U2OS cells are insensitive to the Mcl-1 inhibitor S63845. Fractional viability (FV) measured after 72 hours of drug exposure, measured in FLICK. Data are mean ± SD for n = 3 independent biological replicates. (**D**) Knockout of MCL1 is not lethal in U2OS cells and does not impact the lethality induced by triptolide. (Top) Immunoblot of Mcl-1 protein levels in U2OS cells expressing sgRNA targeting MCL1 or nontargeting sgRNA. (Bottom) Lethal fraction (LF) kinetics between the two genotypes following exposure to triptolide or vehicle (DMSO, 0.1%). LF kinetics were measured in FLICK, and data are mean ± SD for n = 3 independent biological replicates. Protein lysates used for the immunoblot were taken from the same pool of cells used for LF measurements and were collected at the time of drugging (“0 hours”).

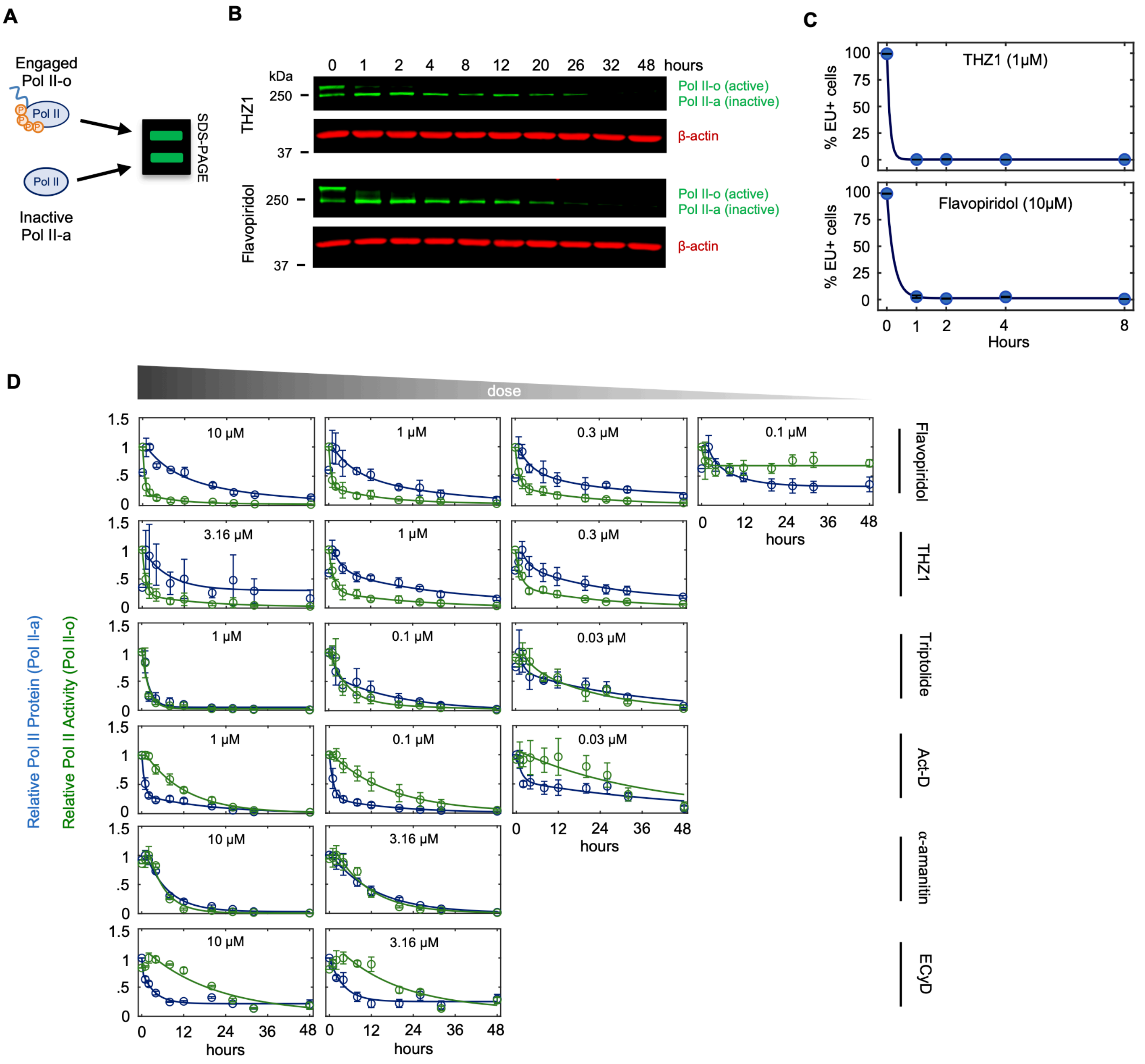

Fig. S9: Variation in kinetics of Pol II inhibition across transcriptional inhibitors. (**A**) Pol II protein primarily exists in two forms. Pol II-o is hyper-phosphorylated along the carboxy-terminal domain (CTD) of the protein and is the actively elongating pool of Pol II. The hypo-phosphorylated form, called Pol II-a, represents all other inactive forms of Pol II, including preinitiation complex (PIC) bound, promoter paused, early pause released, and free Pol II. Pol II-o and Pol II-a can be distinguished by a band shift on a gel. (**B**) Example immunoblot of Rpb1 levels following exposure to two fast acting transcriptional inhibitors, THZ1 (1 μM), and Flavopiridol (10 μM). These drugs rapidly decrease Pol II-o levels. (**C**) EU incorporation into nascent RNA following fast acting inhibitors shown in (B), validating that loss of Pol II-o levels is functionally equivalent to loss of transcription. (**D**) Quantification of Pol II-o (green) and Pol II-a (blue) protein levels across different lethal doses of transcriptional inhibitors. Data were collected using quantitative immunoblotting and fit to a two-phase exponential decay function. Act-D, Actinomycin-D; ECyD, Ethynylcytidine. Data are mean ± SD for n =3 independent biological replicates.

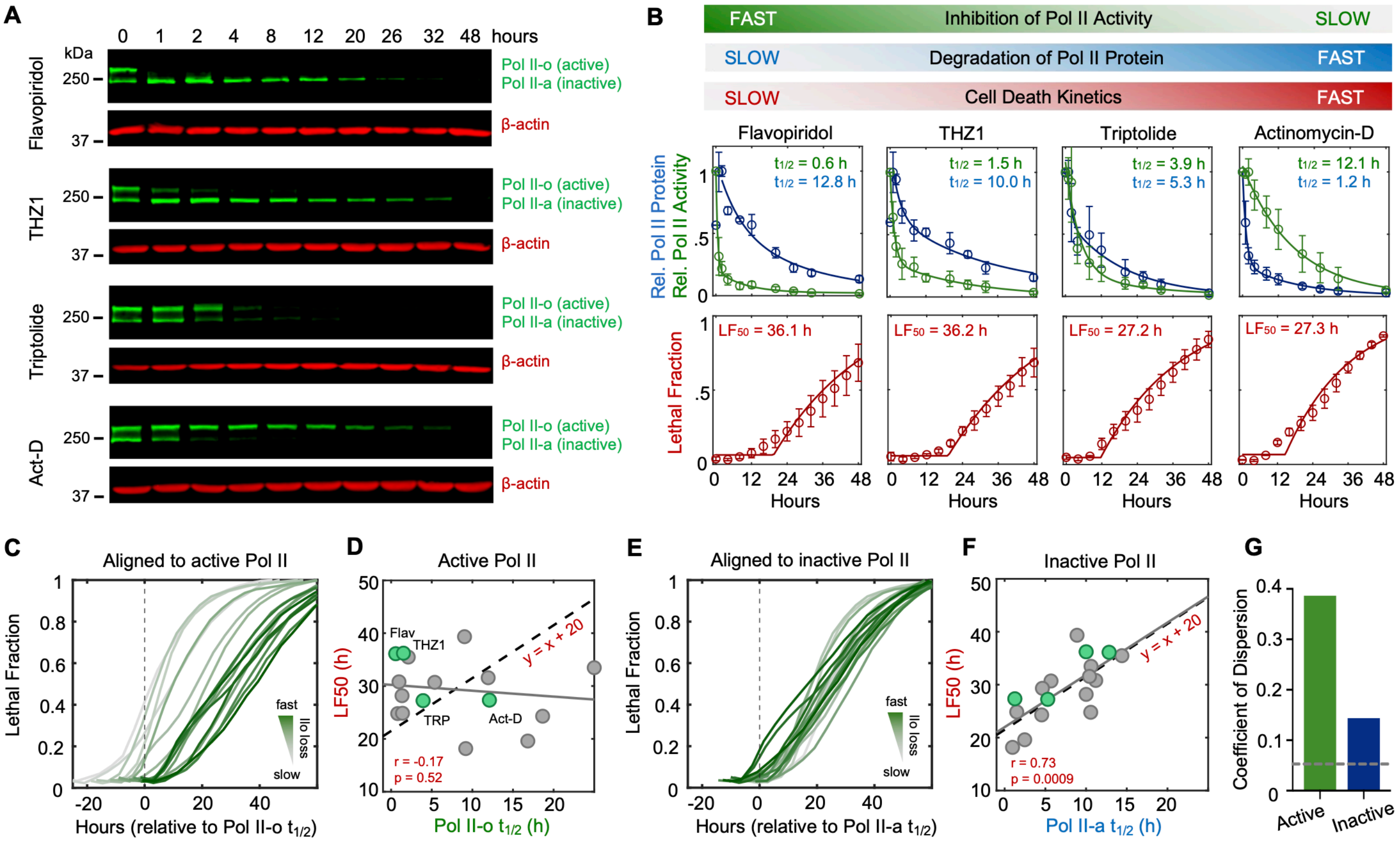

Fig. S10: Loss of Pol II protein – but not loss of Pol II activity – correlates with onset of apoptotic death. (**A**) Immunoblots of total Rpb1 protein following exposure to either flavopiridol (10 μM), THZ1 (1 μM), triptolide (0.1 μM), or actinomycin-D (Act-D, 0.1 μM). Blots are representative of three independent biological replicates. (**B**) (Top panel) Quantification of Pol II-o and Pol II-a decay kinetics from immunoblots described in (A). Data fit to a two-term exponential decay equation. $t_{1/2}$ denotes time to 50% decay. (Bottom panel) Cell death kinetics quantified using the STACK assay. LF_{50} denotes time to 50% max observed death. For both panels, data are mean ± SD, n ≥ 3 independent biological replicates. (**C-D**) Relationship between loss of Pol II activity and activation of cell death. **(C)** Cell death kinetics for 17 unique transcriptional inhibitor drug-dose combinations, aligned to $t_{1/2}$ for loss of active Pol II (dashed vertical line). Cell death was quantified using the STACK assay, and the mean of 3 independent biological replicates measured every four hours is shown. (**D**) For data in (C), correlation between $t_{1/2}$ for loss of active Pol II and time to 50% lethality (LF50). Solid gray line denotes fit to a linear regression model. Black dashed line denotes a direct x = y relationship, shifted by a constant to account for a lag time between drug activity and cell death. **(E-F)** Relationship between loss of inactive Pol II and activation of cell death. **(E)** Same data as (C), except aligned to $t_{1/2}$ for degradation of inactive Pol II (dashed vertical line). **(F)** For data in (E), correlation between $t_{1/2}$ for degradation of inactive Pol II and time to 50% lethality (LF50). (**G**) Coefficient of Dispersion (CoD) of LF_{50} times for cell death kinetics aligned to active or inactive Pol II loss, as shown in (C,E). Gray dashed line denotes the CoD for the "perfect" alignment, defined by arbitrarily shifting each kinetic curve such that maximum alignment is achieved, implemented using the MATLAB function alignsignals with method "risetime".

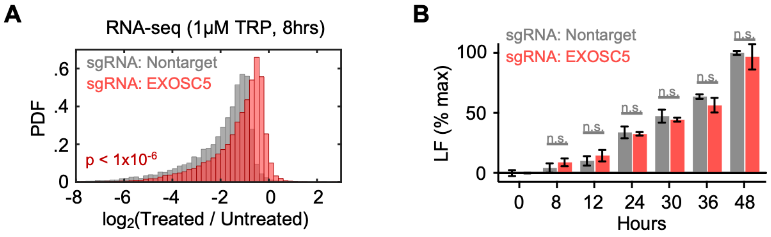

Fig. S11: Effects of triptolide in EXOSC5 knockout cells. (**A**) Histogram of spike-in normalized absolute mRNA fold changes following 8-hr exposure to 1 μM triptolide (TRP) in U2OS cells expressing sgRNA targeting EXOSC5 or nontargeting sgRNA. Two-sided KS test *p* value is shown. (**B**) Comparison of lethal fraction (LF) levels between cell types in (A) at various time points following exposure to 1 μM TRP, measured using the FLICK assay. Data normalized to max LF in cells expressing nontargeting sgRNA. Data are mean ± SD for n = 3 independent biological replicates. Wilcoxon rank sum *p* value shown (n.s. > 0.05).

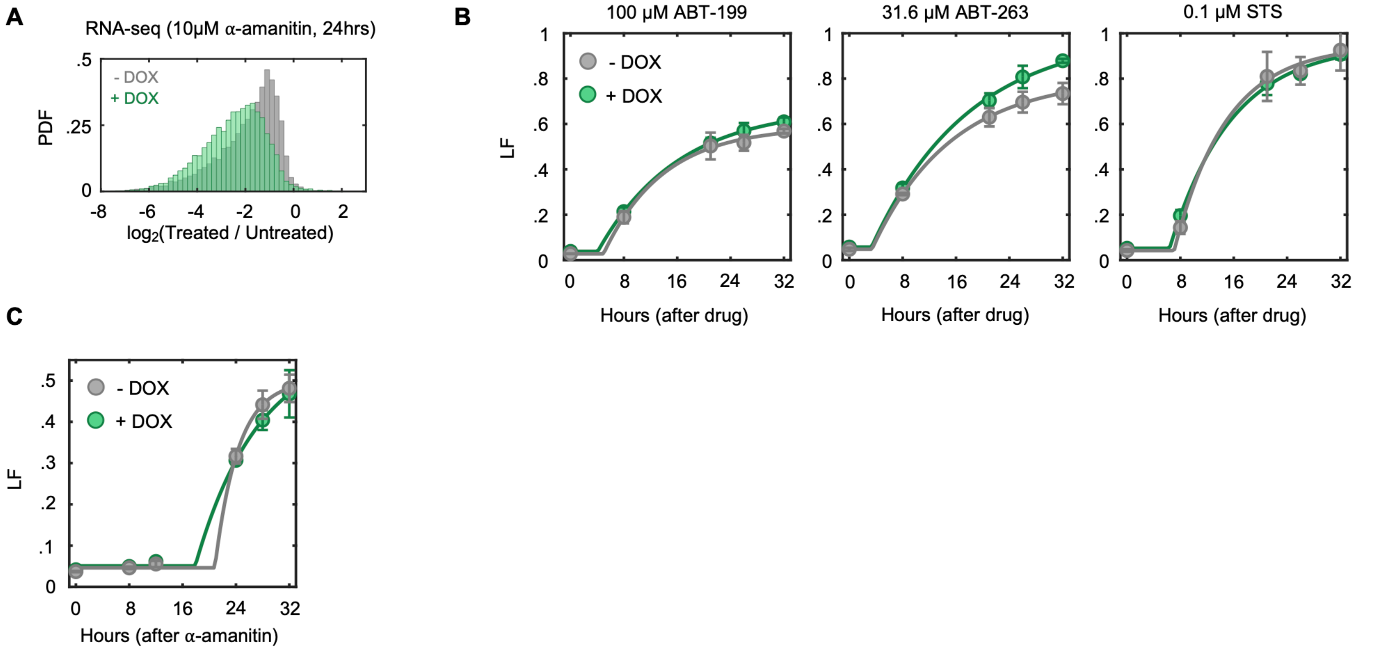

Fig. S12: Characterization of the Pol II switchover system. (**A**) Histogram of spike-in normalized absolute mRNA fold changes following 24-hr exposure to 10 μM α-amanitin. (**B**) Lethal Fraction (LF) kinetics following exposure to general apoptotic agents in Pol II switchover cells with- or without doxycycline (DOX)-induced expression of RPB1-N792D-ΔCTD. LF measured using the FLICK. STS = Staurosporine. (**C**) LF kinetics in cells with- or without expression of a α-amanitin sensitive *RPB1*-ΔCTD transgene, treated with 10 μM α-amanitin and measured using FLICK. For (B-C), mean ± SD shown, n = 3 independent biological replicates.

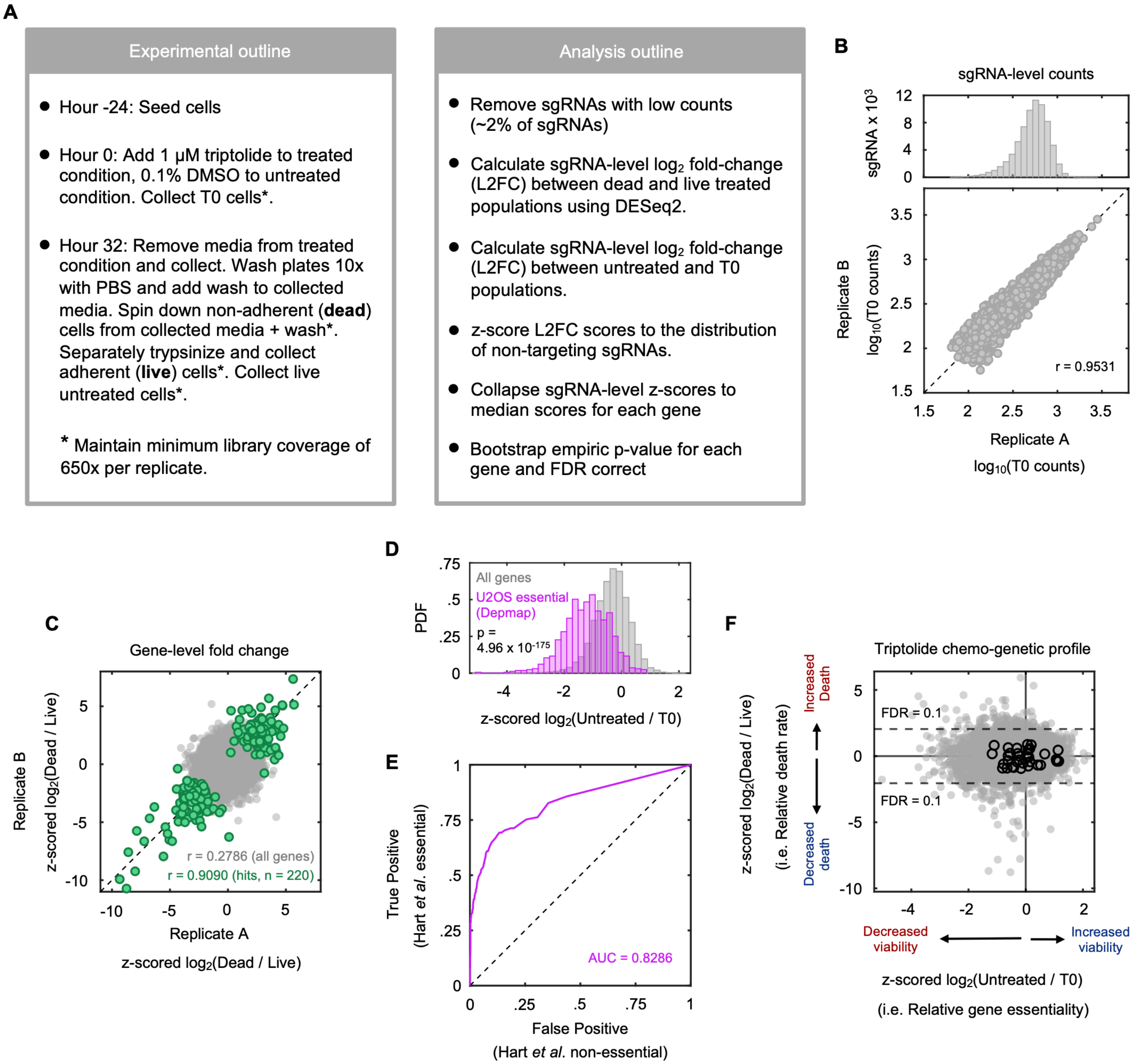

Fig. S13: Chemo-genetic profiling strategy and quality assessment. (**A**) Overview of experimental and analytical process for the chemo-genetic screen. (**B**) (Top) Representative example sgRNA count distribution for replicate A of the T0 condition. (Bottom) Representative example correlation of sgRNA counts between two replicates of the same condition. Dashed line, x = y. Pearson correlation coefficient is shown. (**C**) Correlation of gene-level L2FC scores between two replicates treated with triptolide. (**D**) Distribution of gene-level L2FC scores for essential genes versus all genes in untreated vs T0 comparison. Two-sided KS test *p* value is shown. (**E**) ROC curve depicting the sensitivity and specificity of the untreated versus T0 screening comparison to classify previously established essential and non-essential genes. (**F**) Conceptual overview of metrics comprising the chemo-genetic profile of triptolide. The x-axis describes the effect a gene knockout has on the viability of a cell in untreated conditions. The y-axis describes the effect a gene knockout has on the cell death rate in the context of triptolide. Scores for all genes (gray, filled), and non-targets (black, empty) are shown.

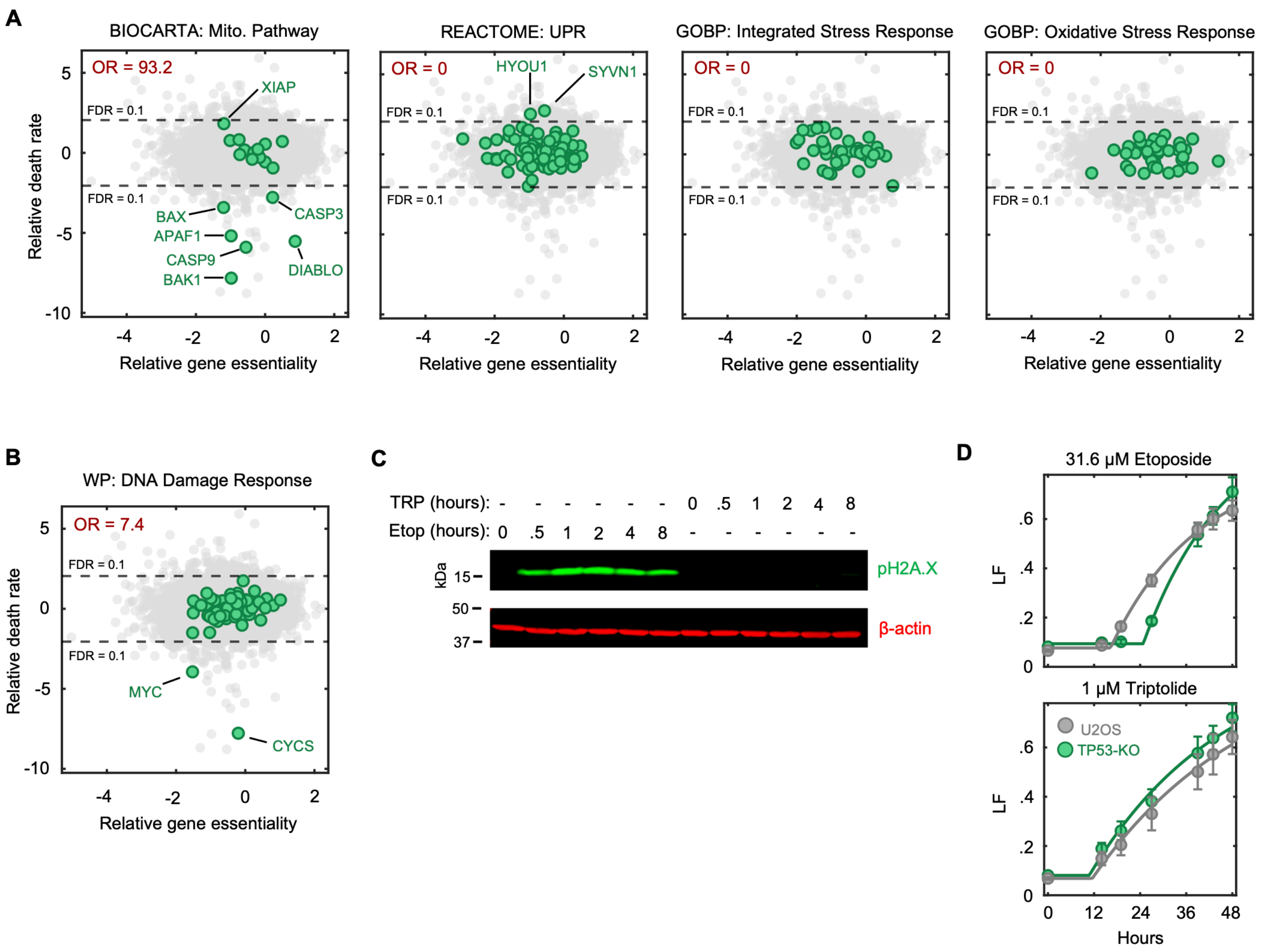

Fig. S14: Triptolide-induced apoptosis does not depend on known stress response pathways. (**A**) Gene-level chemo-genetic profiling data for U2OS cells treated with 1 μM triptolide, highlighting genetic signatures associated with stress response pathways. Signatures defined from the Molecular Signatures Database (MSigDB). The mitochondrial pathway, involved in intrinsic apoptosis, are enriched (far left) among the genes whose knockout decreases triptolide-induced death. The unfolded protein response (UPR), integrated stress response (ISR), and the oxidative stress response are not enriched. Odds ratio for a one-tailed Fisher's exact test is shown (associated adjusted p-values are shown in Fig. 5D). (**B**) As in (A), but for genes associated with the DNA Damage Response (DDR). Two hits, MYC and CYCS, are more generally involved in apoptosis and are not specific to the DDR. (**C**) Immunoblot of phosphorylated H2A.X (Ser139) levels following 31.6 μM etoposide or 1 μM triptolide exposure in U2OS cells, demonstrating that triptolide does not induce DDR signaling, as compared to the DNA damaging drug etoposide. (**D**) Lethal fraction (LF) kinetics following exposure to etoposide or triptolide in U2OS cells or clonal U2OS-TP53 knockout cells, measured in FLICK. Loss of TP53 delays cell death following DNA damage, as previously described (Honeywell, et al., 2024). Loss of TP53 has no effect on cell death following triptolide. Data are mean ± SD for n = 9 independent biological replicates.

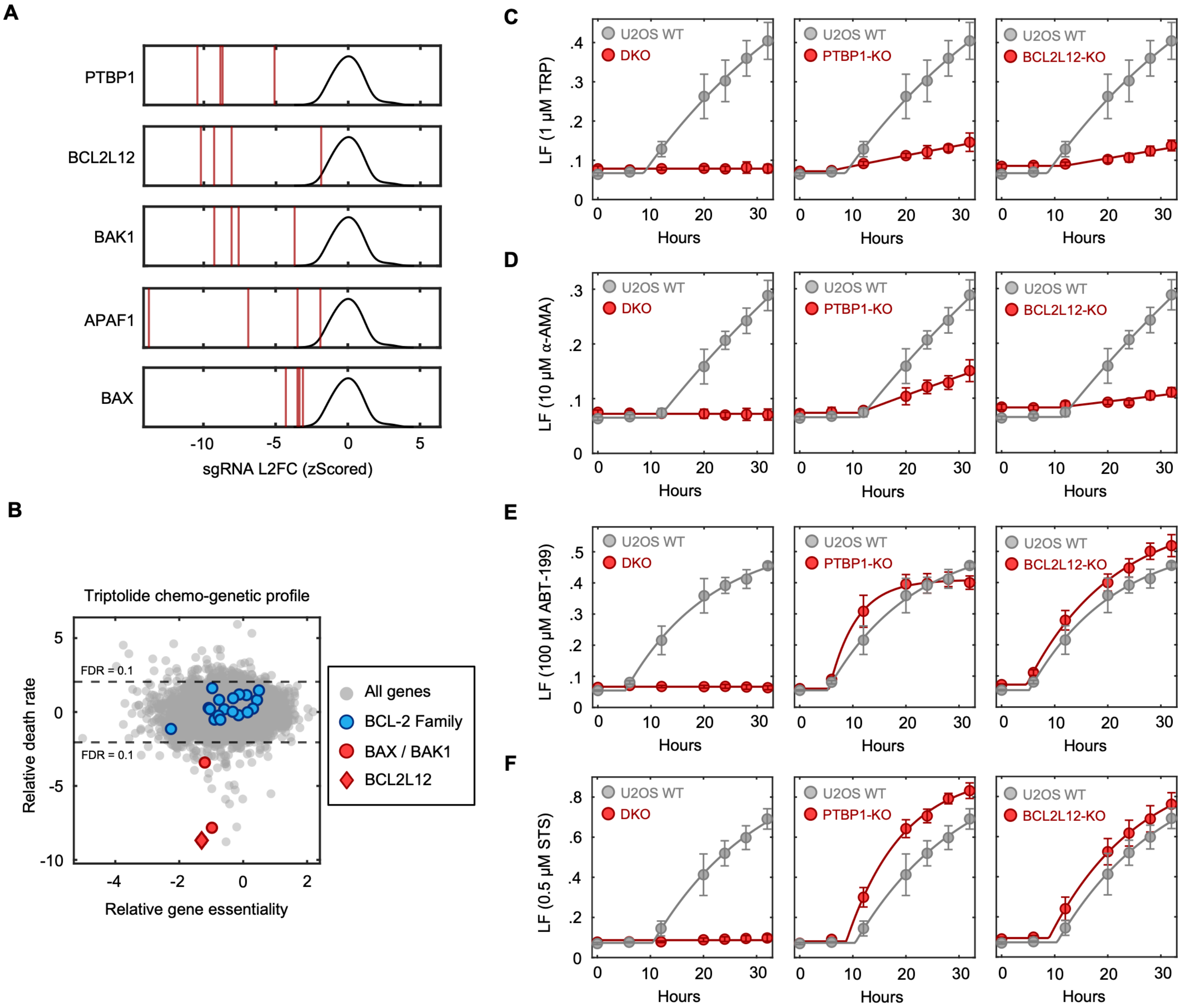

Fig. S15: Validation of PTBP1 and BCL2L12 as unique genetic dependencies of lethality following Pol II degradation. (**A**) sgRNA-level log2 fold changes (dead population / live population) for PTBP1 and BCL2L12 in the context of 1 μM triptolide. BAK1, APAF1, and BAX are core apoptotic regulators and act as controls. Fold changes for 4 sgRNAs per gene (red vertical lines) were z-scored to the distribution of nontargeting guides (black). (**B**) Gene-level chemo-genetic profiling data for U2OS cells treated with 1 μM triptolide. BCL-2 family proteins regulate BAX and BAK1 activity. BCL2L12 is the only BCL-2 family protein identified that modulates cell death following triptolide (apart from BAX and BAK1 themselves). Highlighted BCL-2 family members in blue are MCL1, BCL2L1, BCL2L14, BCL2L10, BCL2A1, BCL2L2, BCL2L13, BAD, BOK, BMF, BCL2, BIK, BCL2L15, BCL2L11, and BID. (**C-F**) Cell death kinetics measured using FLICK in U2OS wild-type cells compared to BAX/BAK DKO (left), PTBP1-KO (middle), and BCL2L12-KO cells (right). **(C)** 1 μM triptolide. **(D)** 10 μM α-amanitin. **(E)** 100 μM ABT-199. **(F)** 0.5 μM Staurosporine. Data are mean ± SD for n = 6 independent biological replicates. Panels (C) and (D) are Pol II-degrading transcriptional inhibitors. Panels (E) and (F) are canonical apoptotic activators.

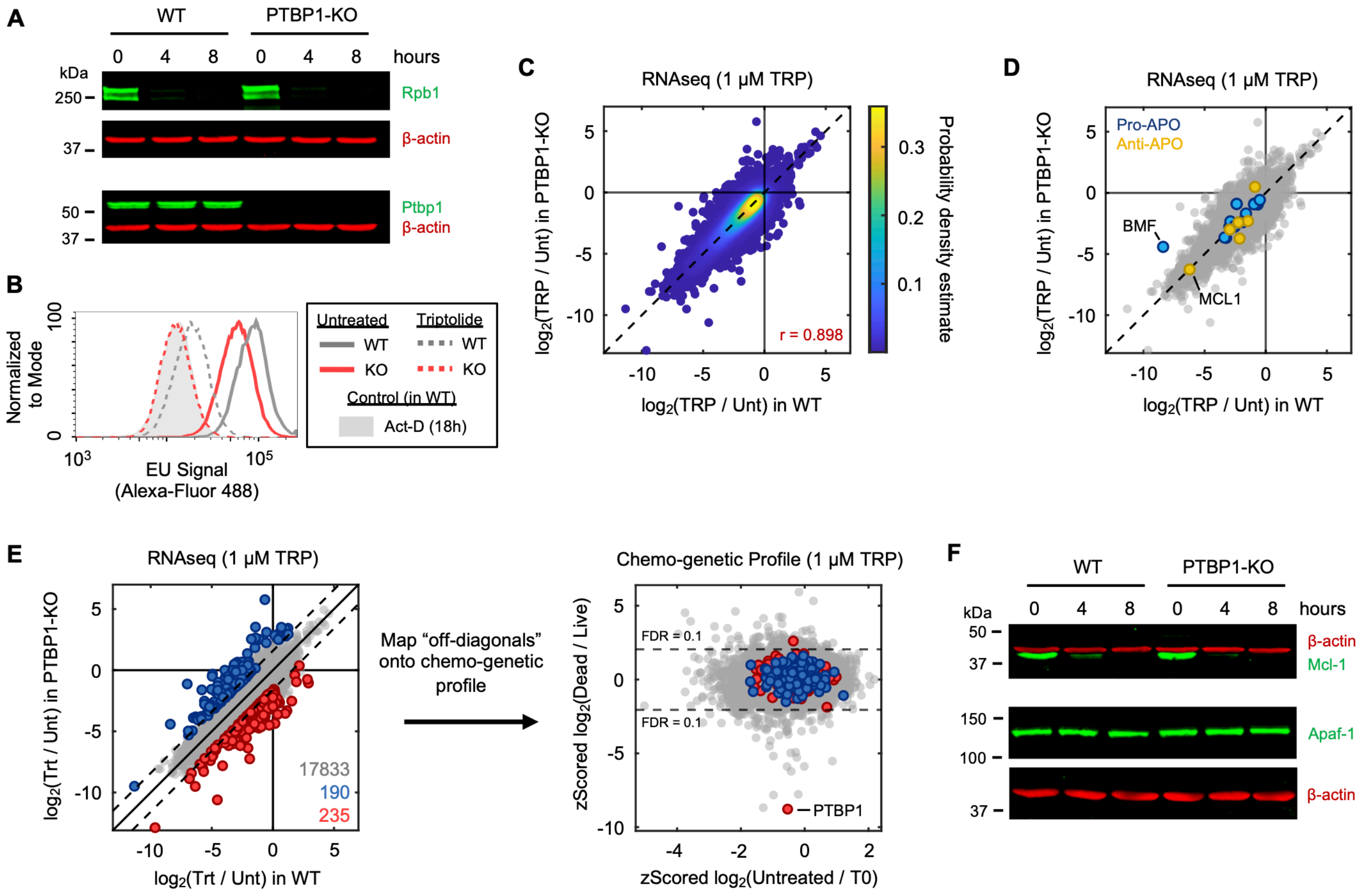

Fig. S16: PTBP1 knockout has no effect on Pol II degradation, loss of nascent transcription, or loss of mRNA, following triptolide exposure. (**A**) Immunoblots for Rpb1 protein levels following exposure to 1 μM triptolide in U2OS and U2OS-PTBP1-KO. (**B**) EU incorporation into nascent RNA in U2OS and U2OS-PTBP1-KO. Cells treated with or without 1 μM triptolide for 8 hours prior to EU labeling. Populations are representative of 3 biological replicates. (**C**) RNAseq comparison of drug-induced transcriptional changes in wild-type (x-axis) and PTBP1-KO (y-axis) cells, measured 8 hours following treatment with 1 μM triptolide. Data were normalized using ERCC spike-ins. Dashed line, x = y. Correlation coefficient shown is for all genes. (**D**) RNAseq data as in (C), highlighting that established anti-apoptotic (Anti-APO) and pro-apoptotic (Pro-APO) regulators show similar expression changes following triptolide in WT and PTBP1-KO backgrounds. Anti-APO: BCL2, MCL1, BCL2L10, BCL2A1, BCL2L2, XIAP. Pro-APO: BCL2L14, BCL2L13, BAD, BOK, BMF, BIK, BCL2L11, BID, BAK1, BAX, APAF1, CASP3, CASP7, CASP8, CASP9, CYCS, DIABLO. (**E**) (Left) RNAseq data as in (C), identifying rare outlier genes whose expression is decreased more (red) or less (blue) in PTBP1-KO cells compared to WT cells following triptolide. The threshold for an “outlier” is depicted by the dashed lines, denoting 2.5 standard deviations from the identity line (x = y). Number of outliers shown. (Right) Mapping of expression outliers onto the chemo-genetic profile for triptolide. (**F**) Immunoblots for Mcl-1 and Apaf-1 protein levels following exposure to 1 μM triptolide in wild-type U2OS cells and U2OS-PTBP1-KO clones.

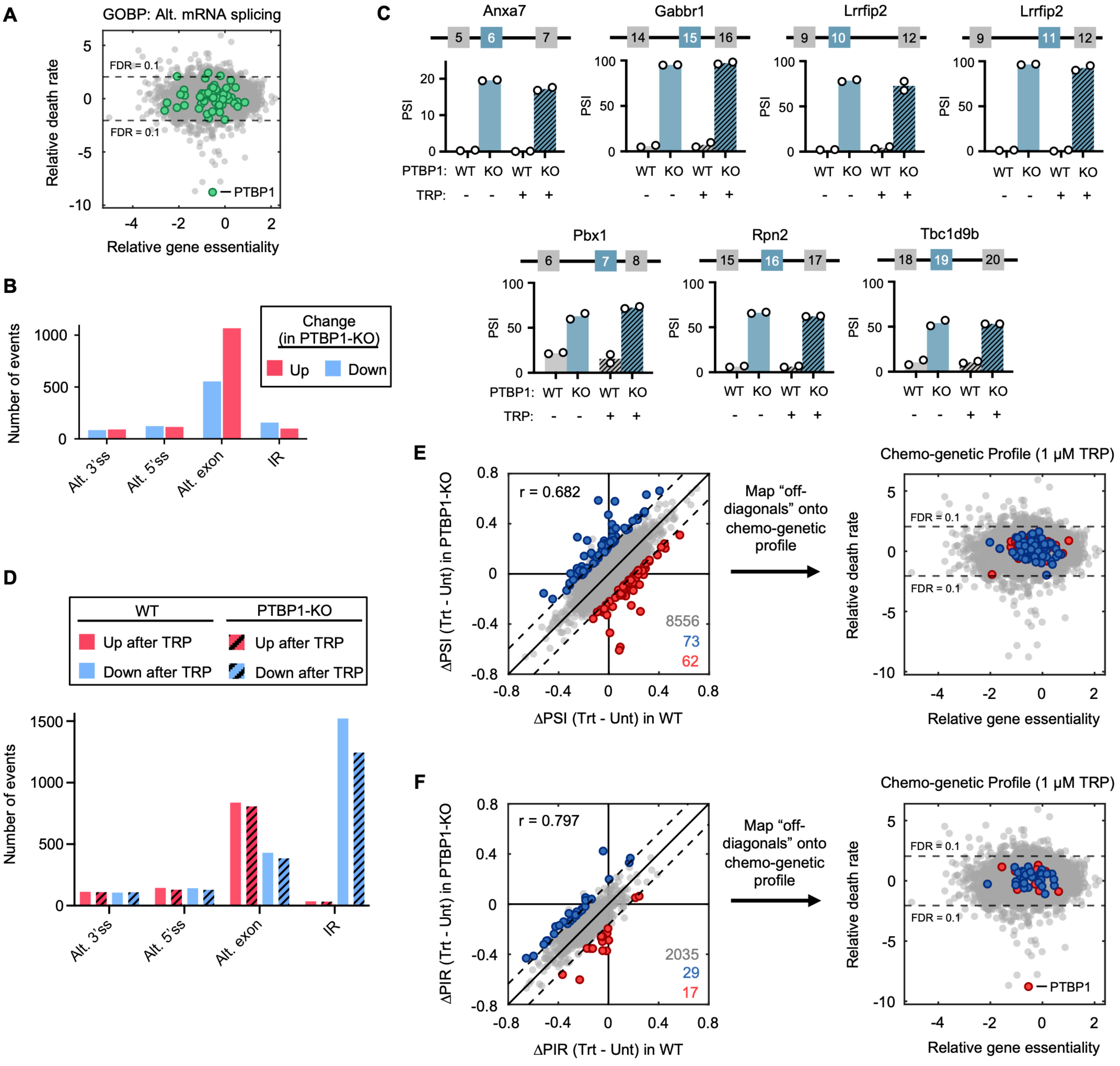

Fig. S17: The role of PTBP1 in triptolide-induced lethality is unrelated to alternative splicing. (**A**) Chemo-genetic profiling data for U2OS cells treated with 1 μM triptolide highlighting genes that regulate alternative splicing, as defined by MSigDB. (**B**) Number and type of significant PTBP1-dependent splicing events identified by comparing U2OS and U2OS-PTBP1-KO RNAseq datasets. (**C**) Previously annotated PTBP1-dependent exon exclusion events. PSI: percent spliced in for the blue colored exon. Data are with- or without 1 μM triptolide for 8 hours. (**D**) Significant splicing alterations induced following 8-hour exposure to 1 μM triptolide in either U2OS (solid bars) or U2OS-PTBP1-KO (dashed bars) cells. (**E**) (Left) Comparison of drug-induced splicing changes in U2OS (x-axis) and U2OS-PTBP1-KO (y-axis) cells, measured 8 hours following treatment with 1 μM triptolide. Data include alternative 3' splice site, alternative 5' splice site, and alternative exon usage. Dashed lines denote 2.5 standard deviations from the identity line (x = y), and off-diagonals are highlighted. (Right) Mapping of outliers onto the chemo-genetic profile for triptolide. (**F**) As in (E), but specifically for intron retention (IR).

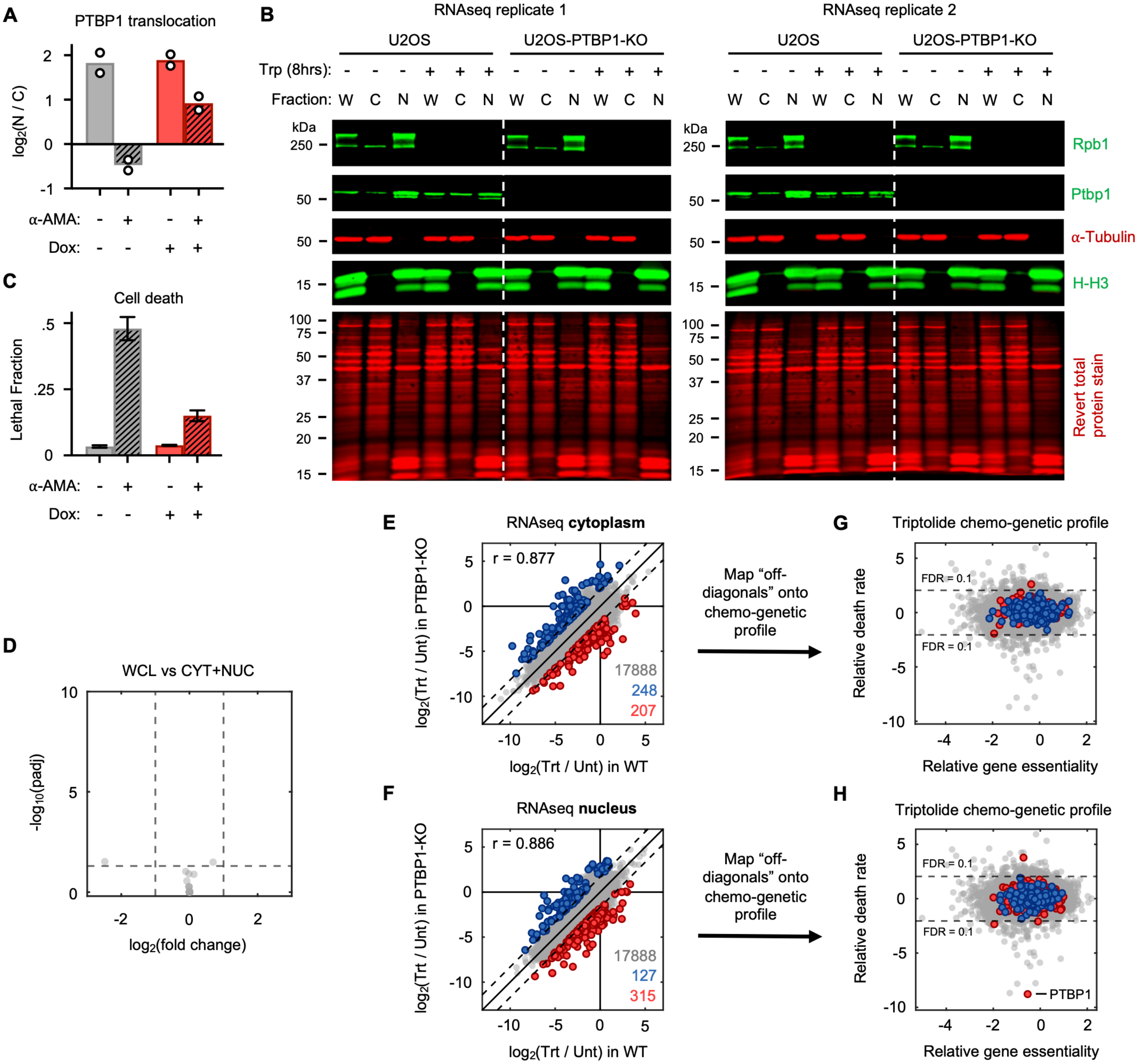

Fig. S18: Evaluation of PTBP1 nuclear export following Pol II degradation. (**A**) Quantification of the nuclear-to-cytoplasmic ratio of PTBP1 protein before and after exposure to 10 μM α-amanitin for 12 hours in Pol II switchover cells, measured using immunoblotting. (**B**) PTBP1 translocation following triptolide (Trp) exposure. These samples were also used for RNA sequencing of nuclear and cytoplasmic fractions. Protein samples were simultaneously obtained from the identical cell lysates used to isolate and sequence mRNA. W: whole cell lysate; C: cytoplasmic fraction; N: nuclear fraction. (**C**) Lethal fraction measured 32 hours after exposure to 10 μM α-amanitin in Pol II switchover cells. (**D**) RNAseq from cytoplasmic and nuclear fractions re-combined to recapitulate the whole cell mRNA extract, validating the quality of the fractionation. (**E**) Comparison of drug-induced expression changes of cytoplasmic mRNA in U2OS (x-axis) and U2OS-PTBP1-KO (y-axis) cells, following 8-hour exposure to 1 μM triptolide. Dashed lines denote 2.5 standard deviations from the identity line (x = y), off-diagonals are highlighted, and Pearson correlation coefficient of all genes is shown. (**F**) As in (E), but specifically for nuclear mRNAs. (**G**) Mapping of outliers from (E) onto the chemo-genetic profile for triptolide. (**H**) As in (G) but for panel (F).

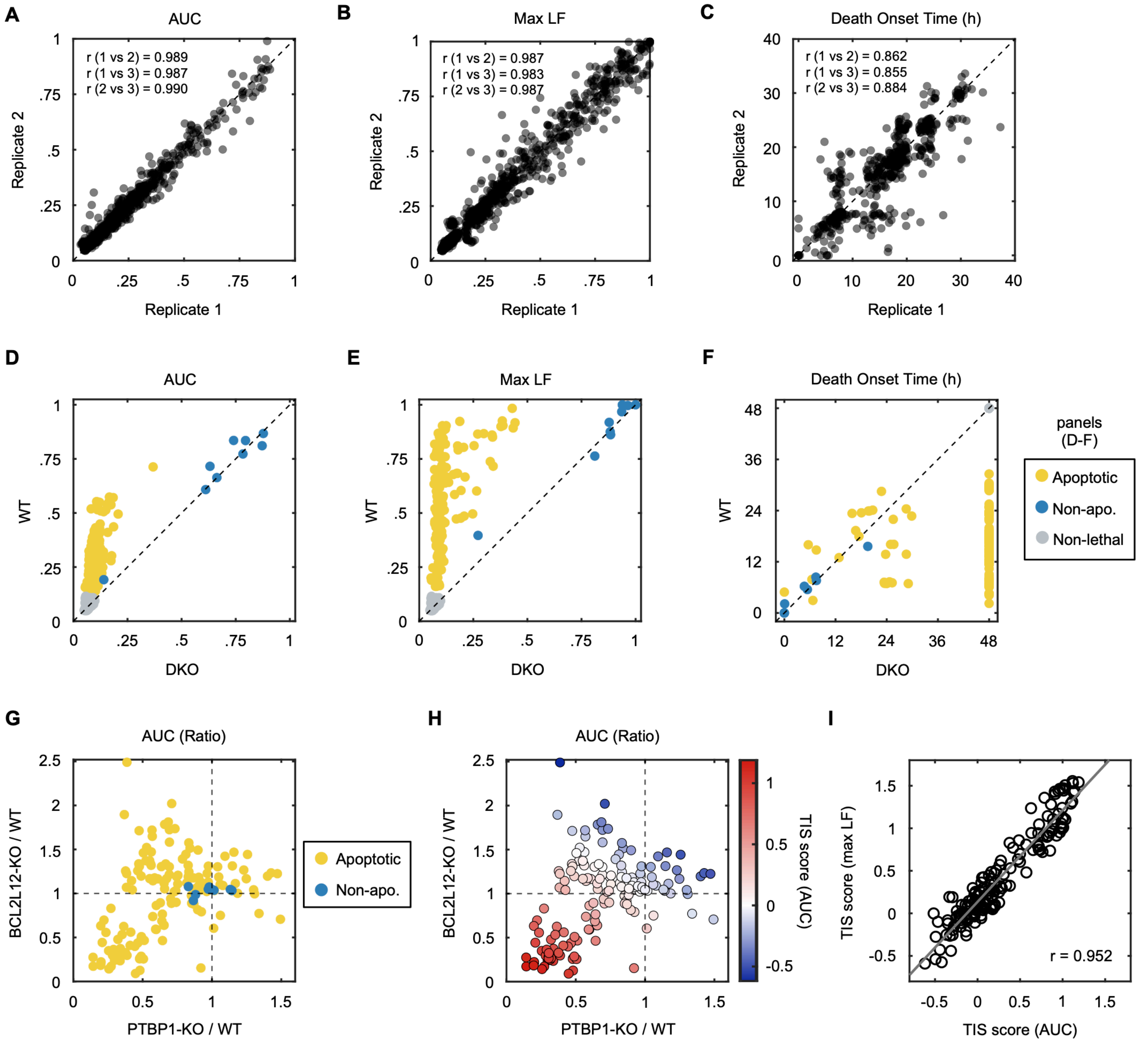

Fig. S19: Evaluation of drug mechanism of killing using temporally resolved death kinetics. (**A**) Quality control for the drug screen shown in Fig. 4. Correlation of AUC values between replicates. Each point represents a single drug-dose-genotype combination, measured using FLICK. Dashed line, x = y. Pearson correlation coefficient for each pair of replicates is shown. (**B**) As in (A), but for the maximum lethal fraction observed at assay endpoint (48 hours). (**C**) As in (A), but for the onset time of death. (**D-F**) Comparison of responses between *BAX/BAK1* DKO cells and U2OS cells for each drug-dose pair. Each condition is classified as apoptotic, non-apoptotic, or non-lethal. (**G**) Comparison of the change in cell death response following knockout of PTBP1 (x-axis) or BCL2L12 (y-axis) for each lethal drug-dose pair. Values of 1 denote no difference from parental U2OS cells. (**H**) Plot depicting how TIS scores vary across the response space. (**I**) AUC is a non-biased metric for generating TIS scores. TIS scores were calculated using max LF in an identical manner as AUC, producing similar results. Linear regression line is shown, along with the Pearson correlation coefficient.

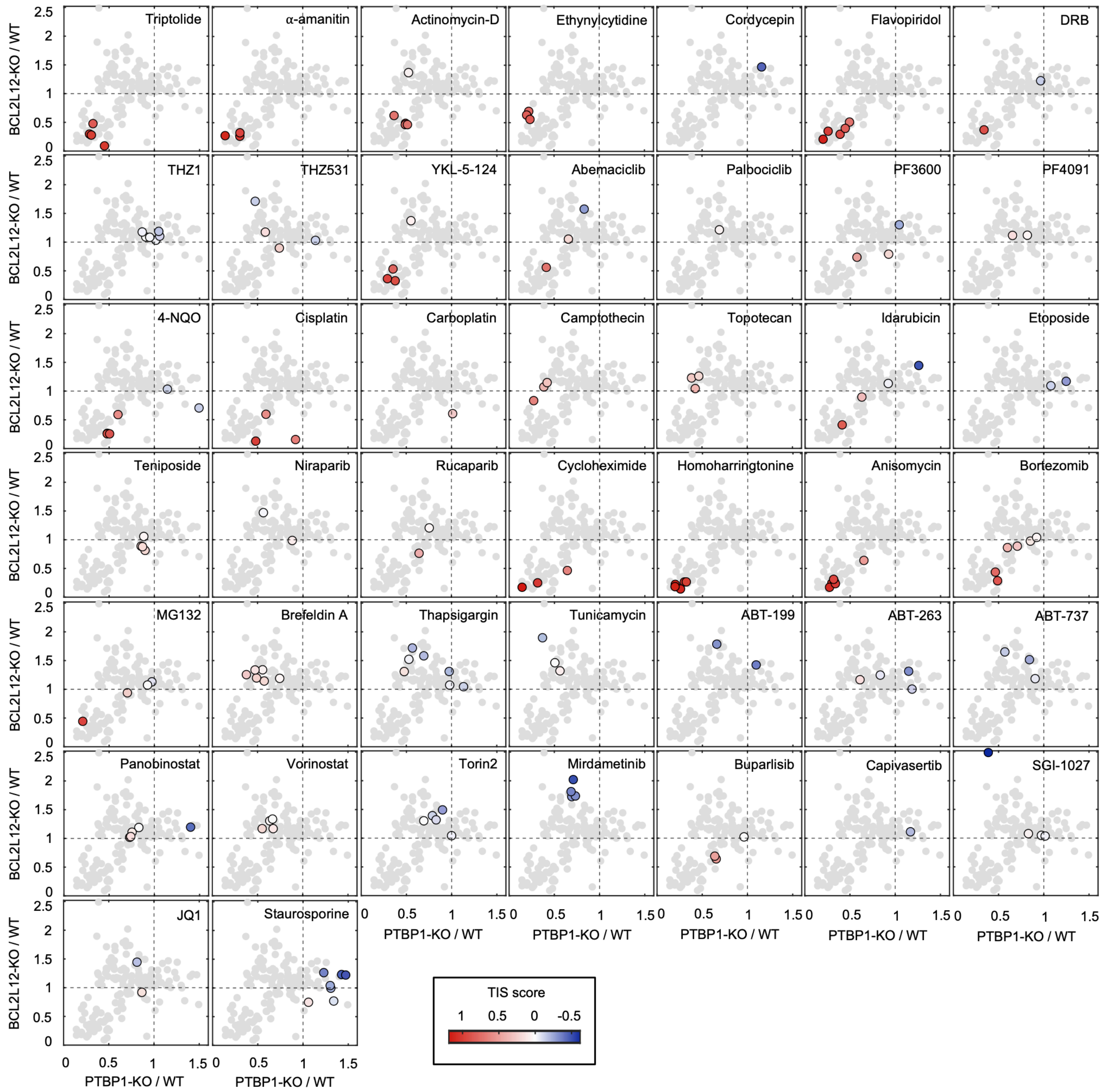

Fig. S20: Evaluation of drug response using TIS score. Map of the functional effect of PTBP1 and BCL2L12 knockout on the cell killing of each drug-dose pair (gray points). For each lethal drug, lethal doses are shown and colored by TIS score. Color bar applies to all panels.

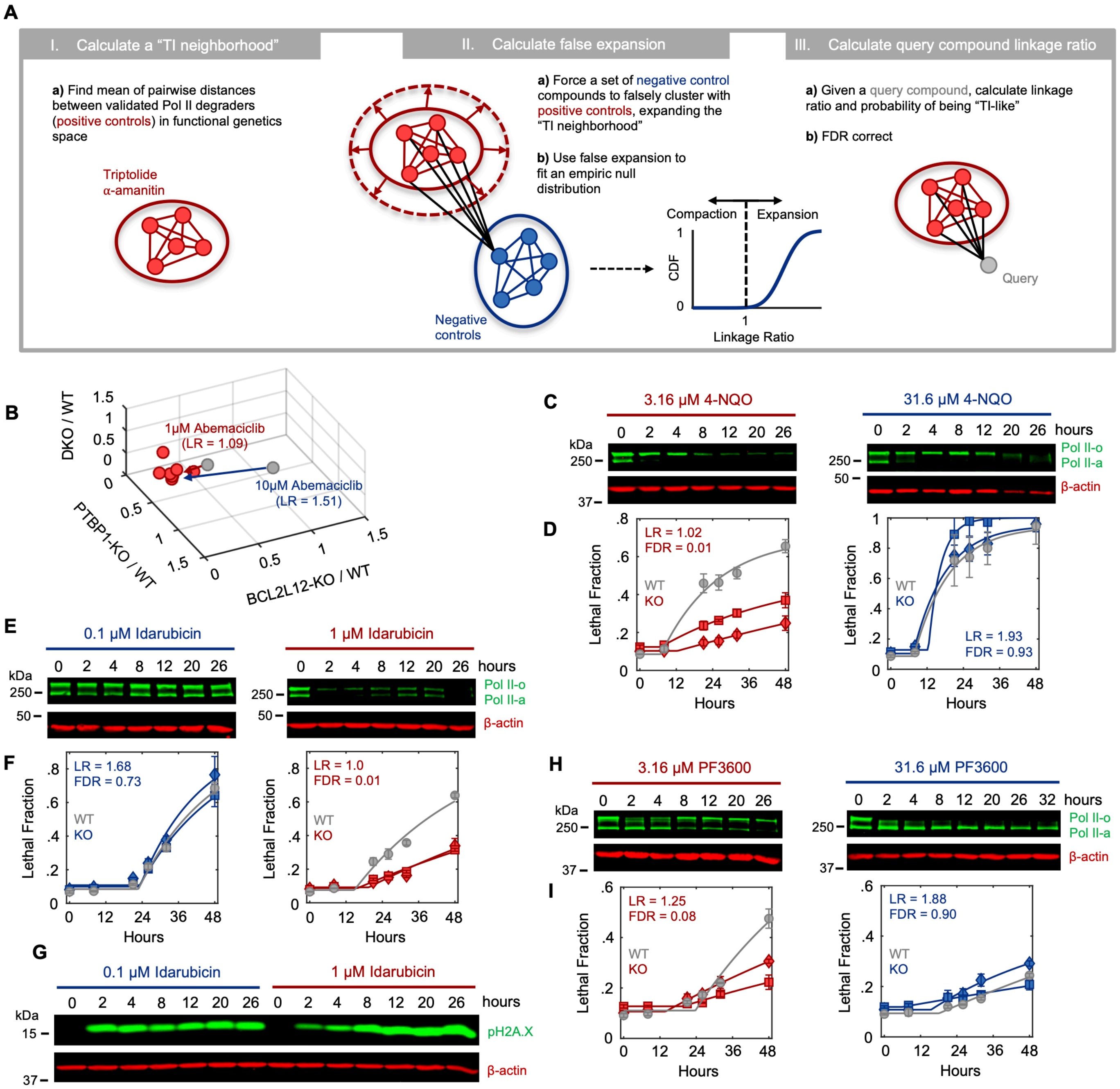

Fig. S21: A statistical classifier to identify "TI-like" compounds. (**A**) Conceptual overview of the probabilistic nearest neighbors-based classification approach (adapted from Pritchard, et al., 2013). (**B**) Classification of Abemaciclib. Triptolide and α-amanitin (positive controls) are shown in red, Abemaciclib (query drug) is shown in gray. 1 μM Abemaciclib is classified as "TI-like", whereas the 10 μM dose is not. Linkage ratios (LR) are shown. (**C**) Immunoblots of Rpb1 protein levels in U2OS cells following exposure to two different doses of 4NQO (3.16 μM left, 31.6 μM right). (**D**) Lethal fraction kinetics for 1 μM Abemaciclib (left) and 10 μM Abemaciclib (right) in U2OS cells (gray circles), PTBP1-KO cells (squares) and BCL2L12-KO cells (diamonds), measured using FLICK. Linkage ratios and associated FDR values are shown. Data are associated with blots above (respectively) in (C). (**E-F**) As in (C-D) for 0.1 μM and 1 μM Idarubicin. (**G**) Similar DDR signaling, as measured by pH2A.X levels in immunoblot, are seen at both doses of Idarubicin shown in (E-F). (**H-I**) As in (C-D) for 3.16 μM and 31.6 μM PF3600. For all panels TI-like drugs are red, Not TI-like are blue.

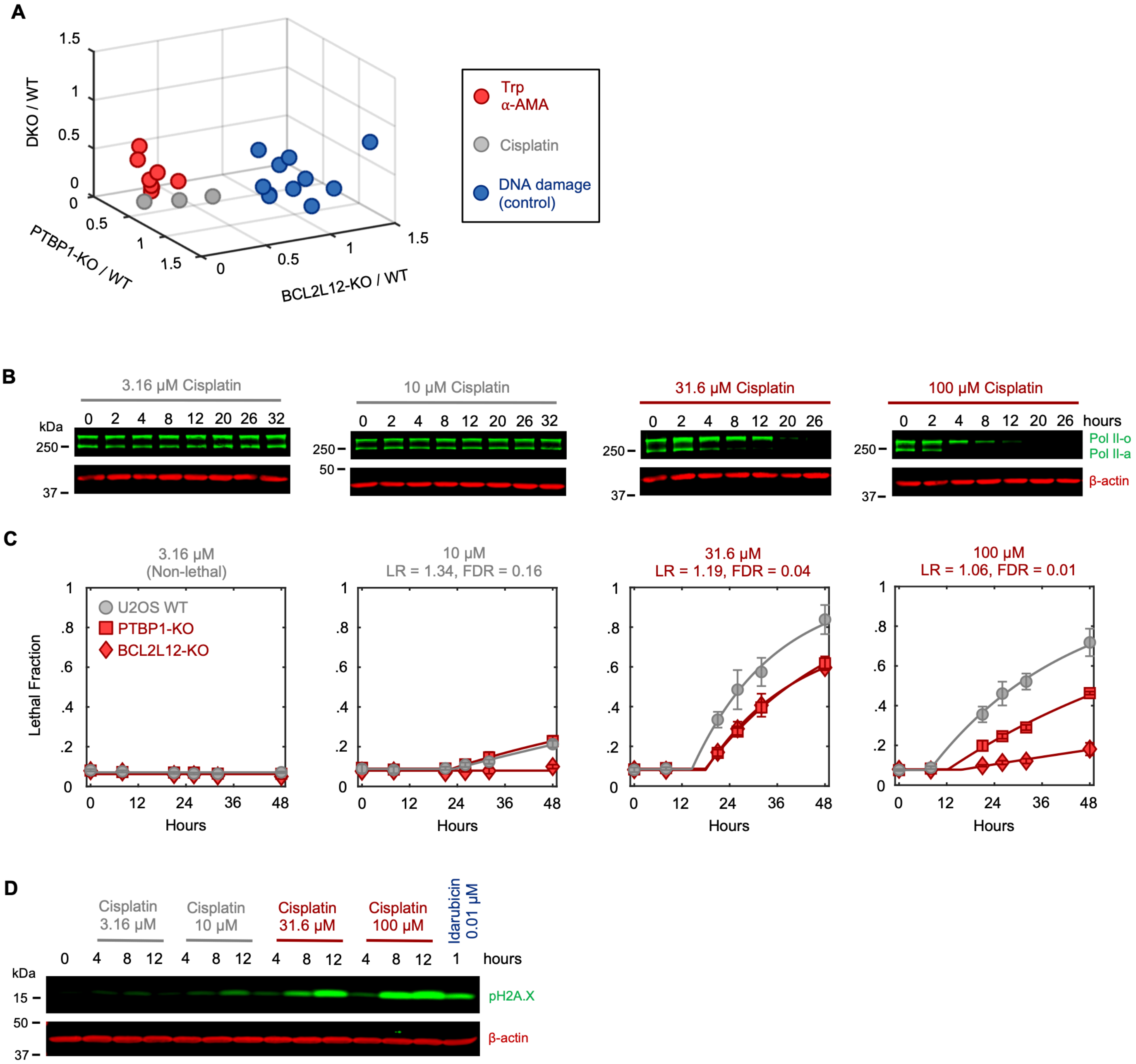

Fig. S22: Lethal doses of Cisplatin require PTBP1 and BCL2L12 for cell killing and degrade Pol II protein. (**A**) Classification of Cisplatin. Triptolide and α-amanitin (positive controls) are shown in red, Cisplatin (query drug) is shown in gray. Other DNA damaging agents are shown in blue (Etoposide, Teniposide, Topotecan, and low-dose Idarubicin). (**B**) Immunoblots depicting Rpb1 protein levels over time following exposure to a range of Cisplatin doses. Images are representative of 3 independent biological replicates. (**C**) Lethal fraction kinetics of Cisplatin in U2OS, PTBP1-KO, and BCL2L12-KO cells, assessed at doses from (B). Linkage ratios and associated FDR values are shown. Data were collected in FLICK, and data are mean ± SD, n = 3 independent biological replicates. (**D**) Immunoblot of p-H2A.X levels across doses of Cisplatin. All doses of Cisplatin that induce appreciable amounts of p-H2A.X signaling are triptolide-like. A low, and non-lethal, dose of the DNA damaging agent Idarubicin is shown for comparison.

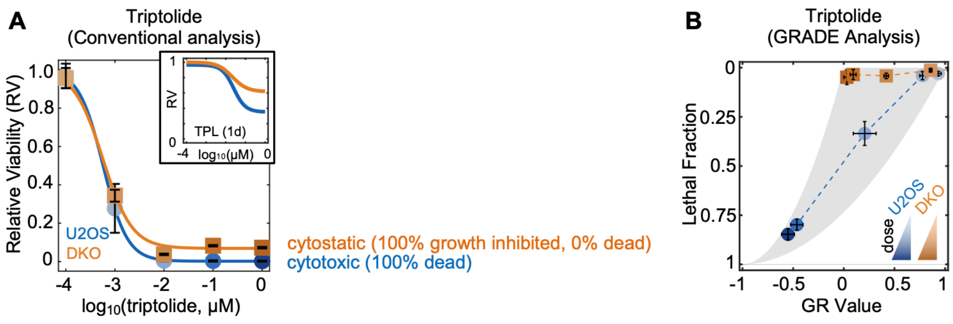

Fig. S23: Conventional analysis metrics are insensitive to cell death and mask the existence of PDAR. (**A**) Triptolide (TPL) sensitivity in U2OS and DKO cells quantified using the conventional measure of drug sensitivity, Relative Viability (RV). RV was measured using the STACK assay 120 hours after drugging, or after 24 hours (inset). (**B**), GRADE analysis to infer the drug-induced growth rate and rate death rate from measurements of the lethal fraction and net population growth rate (GR value). Data for U2OS and DKO cells treated with triptolide for 48 hours, measured using STACK. For all panels with error bars, data are mean ± SD, n ≥ 3 independent biological replicates.
